# Bilberry and bilberry press cake as sources of dietary fibre

**DOI:** 10.3402/fnr.v59.28367

**Published:** 2015-12-11

**Authors:** Anna-Marja Aura, Ulla Holopainen-Mantila, Juhani Sibakov, Tuija Kössö, Mirja Mokkila, Poutanen Kaisa

**Affiliations:** VTT Technical Research Centre of Finland Ltd., Espoo, Finland

**Keywords:** bilberry, press cake, non-digestible carbohydrate, sulphuric acid–insoluble material, microstructure

## Abstract

**Background:**

Dietary recommendations for Nordic countries urge the use of plant foods as a basis for healthy nutrition. Currently, the level of dietary fibre (DF) intake is not adequate. Berries are an elementary part of the recommended Nordic healthy diet and could be consumed in higher amounts.

**Materials and methods:**

Finnish bilberries and a bilberry press cake from juice processing were studied for DF content, carbohydrate composition, and non-carbohydrate fibre content, which was analysed as sulphuric acid insoluble and soluble material. The microstructure of all samples was also studied using light microscopy and toluidine blue O, calcofluor, and acid fuchsin staining.

**Results:**

The total DF contents of fresh and freeze-dried bilberries and the press cake were 3.0, 24.1, and 58.9%, respectively. Most of the DF was insoluble. Only about half of it was carbohydrate, the rest being mostly sulphuric acid–insoluble material, waxy cutin from skins, and resilient seeds. Bilberry seeds represented over half of the press cake fraction, and in addition to skin, they were the major DF sources. Microscopy revealed that skins in the press cake were intact and the surface of the seeds had thick-walled cells.

**Conclusions:**

Bilberry press cake is thus a good source of insoluble non-carbohydrate DF, and could be used to provide DF-rich foods to contribute to versatile intake of DF.

Bilberry (*Vaccinium myrtillus* L.) is a shrub growing on acidic soils in various climates and is native to Eurasia and Northern America. In the Nordic countries, bilberries are among other forest berries and part of the national food culture. In addition, wild bilberries have been considered as superfruits and there has been a hype surrounding bilberry and blueberry, based on their high content of anthocyanins (1–4), the health benefits of which have been reviewed recently by Pojer et al. (5).

The average Danish intake of berries is 5 g/day, whereas the recommendation according to the New Nordic Diet is 50–100 g/day (6). The Norwegian recommendation is two portions a day for fruits and vegetables, one portion being 1–2 dl of fresh fruits and vegetables (7). In Finland, only 14% of males and 28% of females (25–64 years) consume berries. The berry consumer's average intake of berries in Finland is 56 g/day for women and 66 g/day for men, when average consumption of berries among all consumers (25–64 years) is only 10 g/day for men and 16 g/day for women (8). According to the same study, the mean amount of berry-based products (calculated as fresh berries) in diets is 18 g/day for men and 25 g/day for women (25–64 years).

Fresh berries are seasonal food, and the consumption is highest during summer. In Finland, wild berries are harvested both commercially and domestically. Bilberry and lingonberry (or cowberry) (*Vaccinium vitis-ideae* L.) are commercially the most important wild berries in Finland. The average annual bilberry yield is 184 million kg, varying from 92 to 312 million kg. Only around 5–6% of the total yield of bilberries is collected (9). Domestic and industrial processing enables their consumption even in other times of the year. In addition to frozen berries, berry jams and juices/nectars and dried berries are used. Bilberries are often compared with cultivated blueberries which have been branded as ‘a superfruit’ based mainly on their high content of anthocyanins and antioxidant activity. The pulp from wild bilberries is different from blueberries; bilberries have higher anthocyanin content than blueberries and there are also some differences in the contents of flavonols and hydroxycinnamic acids (5, 10, 11). Blueberries have a minor role in berry cultivation and consumption in Finland. Their cultivation area is only 76 hectares (year 2013) and annual yield is 0.09–0.13 million kg (12).

Wild berries are considered a natural part of a healthy diet in the Nordic countries (6, 7, 13, 14). Adamsson et al. (14) describe a healthy Nordic diet as a plant-based diet with a dietary pattern associated with decreased morbidity and mortality. In a large randomised Nordic study (Sysdiet), a healthy Nordic diet included whole grain products, berries, fruits and vegetables, rapeseed oil, three fish meals per week, and low-fat dairy products. This dietary pattern improved the lipid profile and had a beneficial effect on low-grade inflammation in people having markers of metabolic syndrome (13). A healthy Nordic diet with vegetables, bilberries, fatty fish, and whole grain products improves endothelial dysfunction, lipid profile, and low-grade inflammation in obese individuals or those suffering from features of metabolic syndrome at risk of diabetes (13, 15–17). Berries are a source of dietary fibre (DF) and other nutrients, and they can have an impact on cardiovascular health (18). Particularly bilberries have shown to reduce low-grade inflammation in individuals suffering from metabolic syndrome (19).

Botanically, bilberry (diameter 7–9 mm) represents a fleshy fruit type, which is developed of five united carpels or syncarpous flower. The juicy berry pulp or pericarp surrounding the seeds consists of thin epicarp tissue as the outermost layer, and of watery mesocarp and endocarp tissues towards the centre of the berry. The seeds are elongated and ca. 1 mm long. They reside in endocarp in five locular spaces which are radially and symmetrically separated by partitions. Each locule can contain 10–50 ovules or immature seeds (20, 21). In a study by Nuortila et al. (22), up to 70 mature seeds were detected per single bilberry.

According to the scientific opinion of European Food Safety Authority (EFSA), DF consists of non-digestible carbohydrates and lignin (23). In the current European Union definition, DF is defined as carbohydrate polymers with three or more monomeric units which are neither ingested nor absorbed in the human small intestine. In addition, lignin and other phytochemicals, such as waxes, saponins, cutin and phytosterols, are considered as fibre, when associated with carbohydrate polymers (24). DF content in berries varies between 1 and 7% based on fresh weight (25), and is often assessed as alcohol-insoluble solids (AIS). AIS content in blueberries is 3.6–4.5 g/100 g fresh berries (4). Bilberries contain pectin, hemicellulose, and cellulose, the hemicellulose being mostly xylan (26). In industrial bilberry juice manufacturing, a press cake with high DF content is produced. The AIS content of bilberry press cake has been reported to be 37 g/100 g fresh berries (26, 27). Most of the hemicellulose and cellulose is found in the press cake (26, 28). Therefore, this ingredient could be a good source of bilberry DF, for example, in snacks and baked goods.

The aim of the current study was to characterise DF components in fresh bilberry and dried bilberry press cake, and to evaluate the potential of bilberries and berry products as a source of DF in the Nordic countries.

## Materials and methods

### Raw materials

Commercial frozen Finnish bilberries (*V. myrtillus* L., brand Pirkka, Kiantama Ltd., Suomussalmi, Finland) were purchased from a local store, freeze-dried, and ground with a laboratory mill trough 0.5 mm screen (ZM300, Retsch GmbH, Haan, Germany) before analyses. The press cake from bilberry juice was produced by Kiantama Ltd. (Suomussalmi, Finland) and stored as frozen at −18°C prior to further processing. The press cake was dried in a convective dryer with air circulation at +40°C overnight. The dried press cake was ground for analytical purposes in a fine impact mill (100 UPZ-lb, Hosokawa Alpine AG, Augsburg, Germany) with pin disc grinders at a rotor speed of 18,000 rpm.

**Fig. 1:**
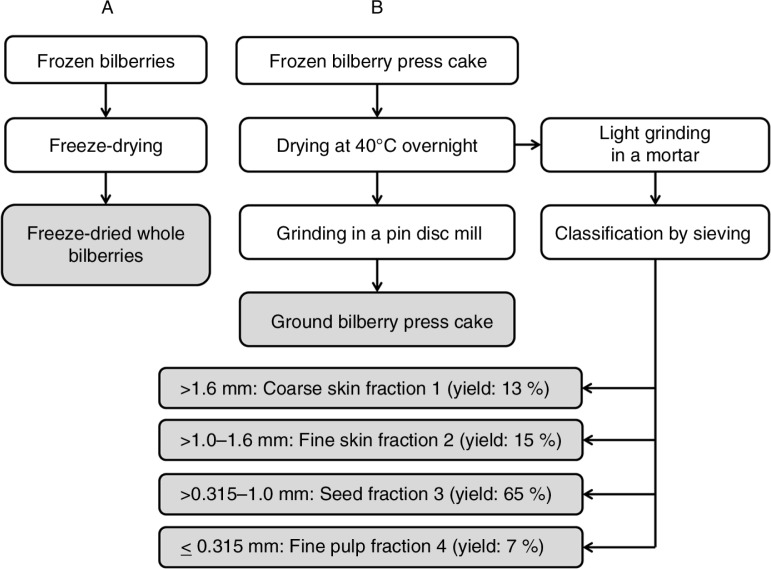
Processing of bilberry, the press cake, and the fractions. The press cake was obtained from a commercial juice manufacturer.

The dried press cake was lightly ground in a mortar for the fractionation to separate the seeds, skin, and fruit flesh without breaking the seeds. The press cake fractions were separated and classified according to the differences in size by sieving, which was carried out using the following sieve mesh diameters: 1.6 mm, 1.0 mm, and 315 µm and a sieve shaker (AS 200 digit, Retsch GmbH, Haan, Germany) for 10 min with an amplitude of 1.0 mm. Bilberry processing scheme is represented in Fig. 1.

### Chemical analyses

DF was determined using an official enzymatic–gravimetric method of Association of Official Agricultural Chemists (AOAC) (29), in which samples are digested with α-amylase, protease, and amyloglucosidase. For the analysis of insoluble DF content, enzyme digestate is filtered, and the residue is washed with warm water and dried. For the analysis of soluble DF content, the combined filtrate is precipitated with alcohol, and filtered, dried, and weighed (29). DF contents were expressed per dry weight and per fresh weight of frozen whole berries. Sulphuric acid–insoluble material (SIM) content was determined gravimetrically from the two-phase hydrolysis with sulphuric acid (70% at 30°C for 1 h and 4% at 121°C for 50 min) using the Klason lignin method for wood components per the National Renewable Energy Laboratory (NREL) standard method by Sluiter et al. (30). Sulphuric acid–soluble material (SSM) concentration in the hydrolysate was detected spectrophotometrically at 215 and 280 nm and calculated using equation (1) described by Goldschmid (31). The extractives were determined gravimetrically after Soxhlet extraction with heptane according to the NREL standard method (30). Moisture content was determined gravimetrically after drying in the oven at 105°C overnight.

1 $${\text{C}}_{\text{Lignin}}=\frac{4.53\cdot ({\text{A}}_{\text{215nm}}-{\text{A}}_{\text{280nm}})\cdot \text{D}}{300}$$

in which C_Lignin_ is the acid-soluble lignin concentration (g/L), A is the absorbance at corresponding wavelength, and D is the dilution coefficient.

To determine carbohydrate content and composition the samples were hydrolysed with sulphuric acid and the resulting monosaccharides were determined by high-performance anion exchange chromatography (HPAEC) with pulse amperometric detection (PAD; Dionex ICS 3000A equipped with CarboPac PA-1 column, Dionex Corporation, Sunnyvale, CA, USA) according to the NREL method (30, 32). The polysaccharide content in the samples was calculated from the corresponding monosaccharides using an anhydro correction of 0.88 for pentoses and 0.90 for hexoses. In order to analyse acidic sugars [methylglucuronic acid, galacturonic acid (GalA), glucuronic acid (GlcA)], samples were pre-treated with endohydrolases (β-glucanase, β-xylanase, β-mannanase, α-arabinosidase, α-galactosidase, β-glucosidase, and β-mannosidase) according to Willför et al. (33) with a modification by using Pectinex Ultra 10,000 nkat/g to enhance hydrolysis of pectin, after which the samples were also analysed by HPAEC with PAD. The pre-column and the separation column (Dionex CarboPac PA-1) were employed at 30°C with a flow rate of 1 ml/min using the following eluents: milli-Q water, 100 mM NaOH, 300 mM Na-acetate/100 mM NaOH, and 300 mM NaOH. The gradient of injecting different eluents was adapted from Willför et al. (33). Results were expressed in g/100 g dry matter (d.m.) unless otherwise stated. Measurements were performed as duplicates.

### Microscopy

Appearance, colour, and surface features of fresh bilberry, dried press cake, and its fractions were examined with Zeiss SteREO Discovery.V8 stereomicroscope equipped with Achromat S 0.5× objective (Carl Zeiss MicroImaging GmbH, Göttingen, Germany) and imaged using an Olympus DP-25 single chip colour CCD camera (Olympus Life Science Europa GmbH, Hamburg, Germany) and the Cel[lcirc]P imaging software (Olympus).

For preparation of semi-thin sections for light microscopy, whole bilberries were lyophilised. Dried press cake and press cake fractions were embedded in 2% (w/v) agar prior to fixation. All samples were fixed in 1% (v/v) glutaraldehyde in 0.1 M Na-K phosphate buffer (pH 7.0), dehydrated in a graded ethanol series, and embedded in hydroxyethyl methylacrylate resin as recommended by the manufacturer (Leica Historesin embedding kit, Leica Microsystems, Heidelberg, Germany). Polymerised samples were sectioned (2 µm sections) in a rotary microtome HM 355S (Microm Laborgeräte GmbH, Walldorf, Germany) using a tungsten carbon knife. The sections were transferred onto glass slides and stained.

For examination of tissue integrity, the sections were stained with aqueous 0.1% (w/v) toluidine blue O (Sigma, St. Louis, MO, USA) for 1 min followed by rinsing with water. Toluidine blue O stained the cell-wall polymers in different shades of blue. Toluidine blue O staining was imaged in brightfield. For localisation of protein and cellulose, the sections were stained with aqueous 0.1% (w/v) acid fuchsin (BDH Chemicals Ltd., Poole, Dorset, UK) in 1.0% acetic acid for 1 min and with aqueous 0.01% (w/v) calcofluor white (Fluorescent brightener 28, Aldrich, Germany) for 1 min. In exciting light (λ_ex_ 400–410 nm; λ_em_>455 nm), intact cell walls stained with calcofluor appear blue and proteins stained with acid fuchsin appear red (34, 35). Starch is unstained and appears black. The samples were examined with an Olympus BX-50 microscope (Olympus Corp., Tokyo, Japan). Micrographs were obtained using a PCO SensiCam CCD colour camera (PCO AG, Kelheim, Germany) and the Cel[lcirc]P imaging software (Olympus).

## Results

### Composition and DF characteristics

The dry matter content of frozen bilberries was 12.5% determined gravimetrically, whereas in dried products it was around 90% (Table 1). Fresh bilberries contained 3.0 g/100 g fresh weight DF, of which most (2.2 g/100 g fresh weight) was insoluble. The DF content according to the AOAC method was 24.1 g/100 g d.m. in whole dried bilberries and 58.9 g/100 g d.m. in the press cake (Table 1). When the pulverised press cake was sieved into different fractions, the major fraction (65% of press cake) was largely composed of bilberry seeds. The DF content of this seed fraction (50.9%) was lower than that of the skin fractions (62–71%). The proportion of insoluble DF of total fibre was 74% in whole bilberries and 88% in the press cake.

**Table 1:** Contents of fibres, lignins, and extractives of bilberries, bilberry press cake, and different bilberry fractions

	Yield	Dry matter	Total dietary fibre	Insoluble dietary fibre	Soluble dietary fibre	Sum of SIM and SSM	SIM	SSM	Extractives	Ash
								
	(%)	(g/100 g)		(g/100 g d.m.)			(g/100 g d.m.)			
Fresh bilberries	100	12.5	3.0^a^	2.2^a^	0.8^a^					
Whole freeze-dried bilberries	100	88.6	24.1	17.8	6.2	16.5	14.9	1.5	7.2	1.40
Ground bilberry press cake	100	94.2	58.9	52.0	6.9	32.7	29.0	3.7	13.8	1.23
Fractions of bilberry press cake (by sieving):										
• Coarse skin fraction: >1.6 mm	13	94.1	62.1	53.7	8.5	36.9	32.4	4.6	10.8	1.21
• Fine skin fraction: 1.0–1.6 mm	15	93.1	71.2	63.2	8.0	35.0	32.3	2.7	8.4	0.97
• Seed fraction: 0.315–1.0 mm	65	94.6	50.9	46.4	4.5	26.7	23.6	3.0	22.7	1.71
• Fine fraction: ≤0.315 mm	7	93.1	68.3	60.6	7.7	33.9	30.5	3.4	8.1	1.49

SIM, sulphuric acid–insoluble material; SSM, sulphuric acid–soluble material.

a Values g/100 g fresh weight.

Fresh bilberries contained 3.3 g/100 g fresh weight for total dietary fibre and 2.6 g/100 g fresh weight for insoluble dietary fibre. Source: National Institute for Health and Welfare. Fineli – Finnish Food Composition Database. www.fineli.fi (36).

The amount of non-carbohydrate materials was evaluated by analysing the sum of SIM and SSM (Table 1). On average, the amount of SIM+SSM was 16.5 g/100 g d.m. in whole freeze-dried bilberries and 32.7 g/100 g d.m. in the press cake, where it varied between 26.7 g/100 g d.m. (seed fraction) and 36.9 g/100 g d.m. (coarse skin fraction). The content of extractives was 7.2 g/100 g d.m. in whole bilberries and 13.8 g/100 g d.m. in the press cake. The seed fraction had the highest content of extractives (22.7 g/100 g d.m.) and the fine skin fraction had the lowest content of extractives (8.1 g/100 g d.m.).

Monosaccharide composition was analysed after acid hydrolysis. The total amount of monosaccharides was similar in berries and press cake (43–45 g/100 g d.m.). Of the press cake fractions, the seed fraction had lower carbohydrate content (36 g/100 g d.m.) than the skin fractions. In all samples, the most abundant monosaccharide was glucose, followed by xylose, GalA, galactose, fructose, and arabinose. The largest differences between whole berries and press cake were in glucose content which was higher in berries (30 g/100 g d.m.) than in press cake (22 g/100 g d.m.), and xylose content, which was lower in berries (4.2 g/100 g d.m.) than in press cake (9.1 g/100 g d.m.). The amounts of all other monosaccharides besides glucose and fructose were somewhat higher in press cake than in whole bilberries (Table 2).

**Table 2:** Sugar composition of bilberries, bilberry press cake, and different bilberry fractions

	Rha	Ara	Gal	Glc	Xyl	Man	Fru	MeGlcA	GalA	GlcA	Total mono-saccharides	As poly-saccharides^a^
	(g/100 g d.m.)	(g/100 g d.m.)	(g/100 g d.m.)	(g/100 g d.m.)	(g/100 g d.m.)	(g/100 g d.m.)	(g/100 g d.m.)	(g/100 g d.m.)	(g/100 g d.m.)	(g/100 g d.m.)	(g/100 g d.m.)	(g/100 g d.m.)
Whole freeze-dried bilberries	0.3	1.5	2.0	30	4.2	0.6	1.9	0.1	3.8	<0.1	45	40
Ground bilberry press cake	0.5	2.2	3.1	22	9.1	1.2	0.9	<0.1	4.0	<0.1	43	39
Fractions of bilberry press cake (by sieving):												
• Coarse skin fraction: >1.6 mm	0.4	2.3	3.4	20	7.0	1.3	1.0	<0.1	5.2	<0.1	41	37
• Fine skin fraction: 1.0–1.6 mm	0.4	1.4	2.5	22	14.0	0.9	0.1	<0.1	4.3	<0.1	46	41
• Seed fraction: 0.315–1.0 mm	0.4	2.0	2.3	18	10.2	1.1	0.1	<0.1	2.3	<0.1	36	33
• Fine fraction: ≤0.315 mm	0.4	1.8	3.2	23	8.0	1.5	0.2	<0.1	4.8	<0.1	43	39

Rha, rhamnose; Ara, arabinose; Gal, galactose; Glc, glucose; Xyl, xylose; Man, mannose; Fru, fructose; MeGlcA, methyl-glucuronic acid; GalA, galacturonic acid; GlcA, glucuronic acid.

a Calculated using coefficient 0.90 for hexoses and 0.88 for pentoses (arabinose and xylose).

### Appearance and microstructure

**Fig. 2:**
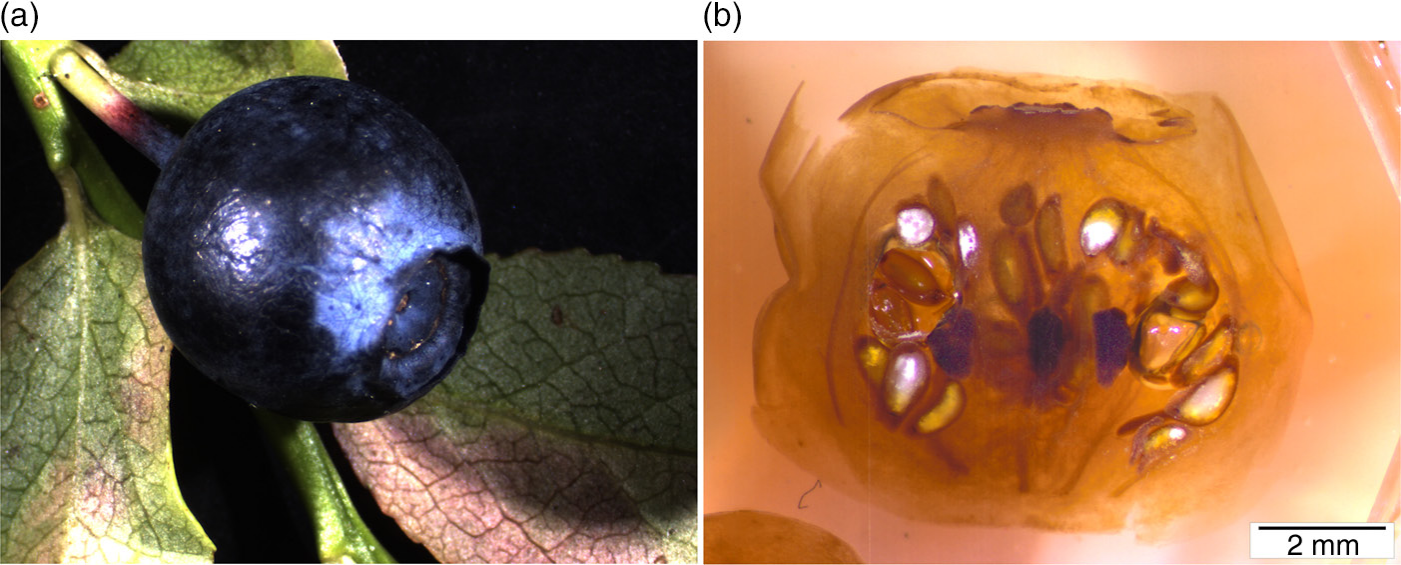
Stereomicroscope images of fresh bilberry (a) and the cross-cut of freeze-dried, resin-embedded bilberry (b).

In appearance, bilberry is a spherical, blue to black–blue berry with a shiny surface (Fig. 2a). On the opposite side to the peduncle or flower stalk, a round structure as the remnant of the corolla is observed. Seeds were concentrated in the middle of the berry surrounded by the layer of fruit pulp (Fig. 2b).

**Fig. 3:**
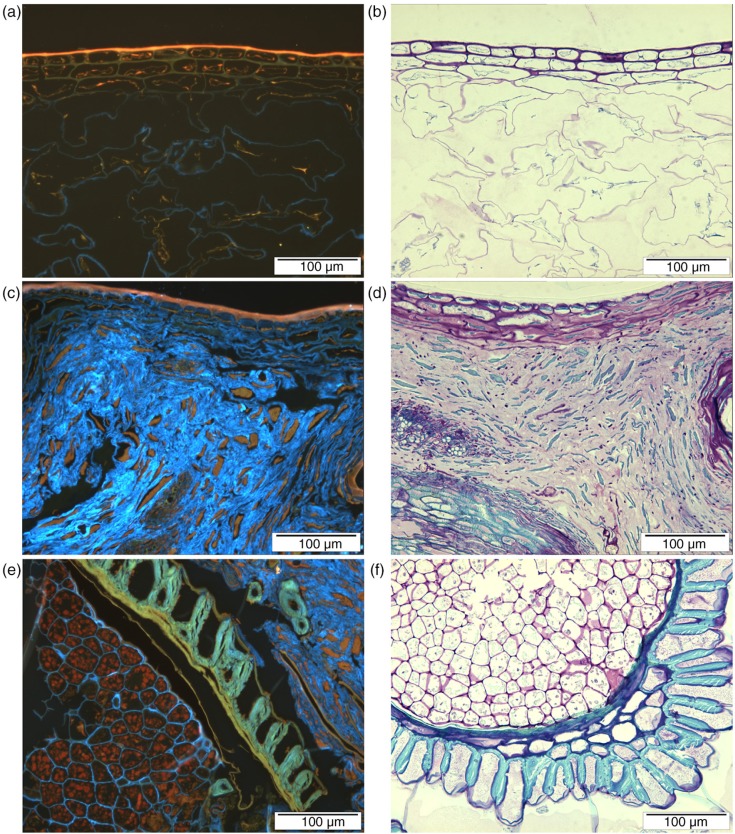
Micrographs showing the structure of fruit pulp in whole, freeze-dried bilberries (a, b), in the press cake (b, c), and structure of bilberry seeds in the whole berry (e, f). Different structures are visualised by calcofluor and acid fuchsin (a, c, e; glucans bright blue and protein red) and by toluidine blue O (b, d, f).

A detailed microstructure of bilberry was revealed by light microscopy (Fig. 3). In whole bilberries, 2–3 cell layers with rather thick cell walls were detected as the outermost structure (Fig. 3a and b). These epicarp cells of skin surrounded the mesocarp tissue (pulp), which consisted of thin-walled cells separated from each other probably because of dehydration during sample preparation. The cuticle, rich in waxes, was detected on the surface of the berry by its strong autofluorescence, which should not be misinterpreted as protein stained by acid fuchsin (Fig. 3a). In the press cake, the most outer cell layers were still distinguishable, but the thin-walled mesocarp cells were heavily compressed (Fig. 3c and d). Cell walls of mesocarp tissue were stained by calcofluor, and some protein was detected inside mesocarp cells by acid fuchsin (Fig. 3a and c). Bilberry seeds were covered by cells having thick secondary walls that were not clearly stained with calcofluor but had strong autofluorescence and formed spike-like structures. (Fig. 3e and f). Even though protein is a minor component in berries, protein was clearly detected inside the seed cells (Fig. 3e).

**Fig. 4:**
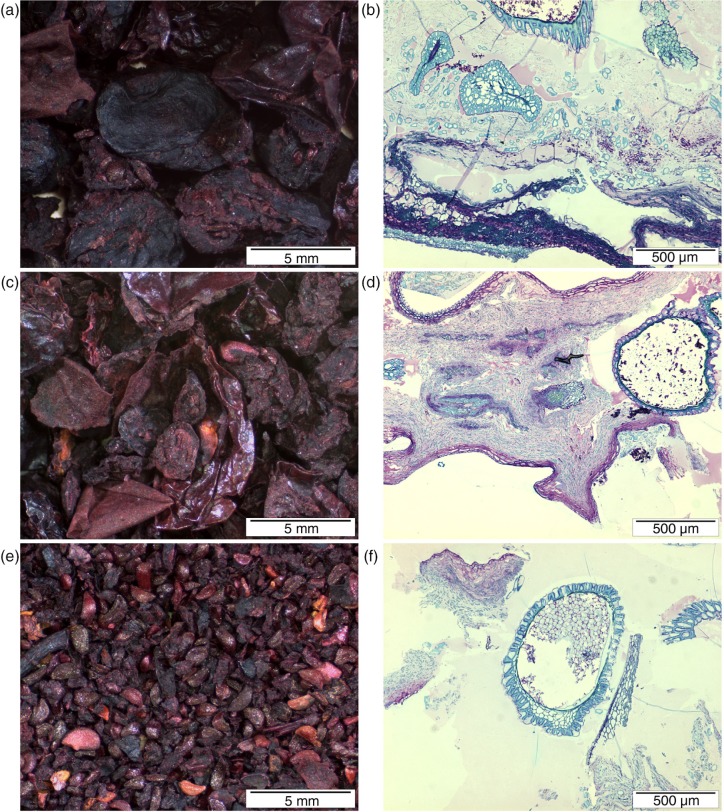
Images showing the appearance (a, c, e) and microstructure visualised by toluidine blue O (b, d, f) of bilberry press cake (a, b) and its coarse skin (b, c) and seed fractions (e, f).

The appearance and microstructure of bilberry press cake, the coarse skin, and the seed fractions are shown in Fig. 4. The press cake clearly consisted of whole berry residues still containing seeds inside the rather intact skins (Fig. 4a and b). In addition, some released thick-walled cells originating from seed surface were observed (Fig. 4b). In the coarse skin fraction, the skins were notably damaged with some seeds present inside the pressed skins (Fig. 4c and d). The seed fraction consisted mainly of seeds, their fragments, and of some pieces of skin and mesocarp (Fig. 4e and f).

## Discussion

Bilberries contained 3.0 g/100 g DF, which was about 25% of their dry weight content (in fresh bilberries, 12.5 g d. m./100 g). This is in line with the values reported in the Finnish Food Composition Database (3.3 g/100 g fresh weight) (36) but slightly less than the values in the USDA database for wild blueberries (4.4 g/100 g fresh weight) (37). The DF content of bilberry press cake was as high as 58.9 g/100 g d.m. The seed fraction made up the majority of the press cake (65%) and contained less DF and more extractives than other fractions.

Higher content of DF in the press cake is because of removal of sugars and other water-soluble compounds in juice pressing. The bilberry press cake was from a commercial juice making process, where berries were treated by an enzyme preparation hydrolysing pectin and hemicellulose. After the enzymatic hydrolysis, juice was compressed with a filter press. Release and removal of soluble sugars by the process also decreased the remaining neutral sugars in the press cake. Buchert et al. (27) described juice extraction using enzymes and characterised anthocyanin recovery in the juices. It is worthy of a note that the press cake still contains anthocyanins because the DF-rich skins contain 20-fold concentration of anthocyanins compared with pulp of bilberry (10). Kaukovirta-Norja et al. (38) patented a process in which phenolic compounds (anthocyanins, ellagitannins, and flavonols) of berry press cakes can be enriched up to 20% (w/w) by separating the skin fraction. The use of press cakes in food applications is usually challenging because of their intensive colour and/or taste. Sibakov et al. (39) described in their patent a method to convert the press cakes into finely ground particles, which can be used in liquid food matrices, such as beverages, jams and fruit soups. The reduction of particle size is advantageous because this fibre-rich material normally may be perceived as sandy or fibrous mouthfeel. Small particle size may also enhance availability of anthocyanins from the skins.

In contrast to cereal DF, which is largely carbohydrate of the cell walls, bilberry DF is largely composed of non-carbohydrate material (sum of SSM and SIM), which in the current study made up 68% of total DF. This high amount is probably explained by the contribution of cuticular waxes and cutin. The enrichment of cuticular layers in the skin fractions was confirmed by microscopy in the present study.

SIM and SSM was analysed using the Klason lignin method developed for analysis of lignin in wood material. The Klason lignin method describes non-specifically any sulphuric acid resilient polymers (40), such as cutin in bilberries (41). Cutin in bilberry is a polyester formed from hydroxyl and epoxy-hydroxy fatty acids (C16 and C18 classes) (42, 43). Complex analysis of cutin has been performed using NMR technique, which identifies components in the cutaneous materials in berries (43–45). Cutin forms the major part of the cuticle, which contains also intra- and epicuticular waxes made of very long-chain fatty acids and their derivatives, and phenolic compounds in cutin matrix (46).

Analogous with cutin, condensed catechins are tightly bound to cell-wall polysaccharides in apples by non-covalent interactions, affecting their quantitation (47, 48). The non-carbohydrate nature of bilberry DF also resembles that of grapes, where high lignin contents have been reported, based on Klason lignin analysis (49). Lignin is a phenolic macromolecule, formed by oxidative random radical coupling of mainly coniferyl and sinapyl alcohols (50). Lignin content has been reported to vary in fruits and vegetables in the range of 1.7–9.0%, analysed as acetyl bromide–soluble lignin (51). The challenge in the gravimetric analytical methods, such as Klason lignin, is not differentiating the compounds in the residue, such as lignin and condensed catechins. Non-specific Klason lignin analysis actually does not verify the presence of lignin in fractions of berries. Lignin may truly be present in berry seeds, but indication of its presence would, however, require pyrolysis gas chromatography. According to Prior et al. (52), procyanidin content of bilberries varies from 3 to 8 µg/g d.w. (corresponding to 0.3–0.8 mg/100 g d.w.), respectively. Therefore, the contribution of procyanidins in the sum of SSM and SIM (16.5 g d.w.) in bilberry can be considered small. The non-carbohydrate fibre could also include waxes, to which resilient carbohydrates are attached, and monomeric phenolic compounds, mainly anthocyanins, which are enclosed in this matrix. Both berries and grapes have high contents of smaller molecular weight phenolic compounds. They might therefore have similar roles in diet; berries in the North and grapes as part of the Mediterranean food pattern. Ovaskainen et al. (53) estimated that the mean total intake of polyphenols in Finnish population would be 863 ±415 mg/d. Phenolic polymers, condensed tannins, and ellagitannins would together contribute 140 mg/d (53).

The carbohydrate content of the press cake was 39%, when calculated as polysaccharides. The high contents of glucose, xylose, and GalA in the press cake suggest the presence of corresponding polymers (glucans, xylans, and pectin) as the main carbohydrate constituents of DF. Hilz et al. (26) characterised cell-wall polysaccharides in bilberry press cake after juice production. The AIS content of bilberry press cake was 36 g/100 g dry berry press cake (26). In the bilberry press cake of the current study, the total monosaccharide content was 43 g/100 g d.m. without alcohol precipitation. The difference is likely because of the removal of alcohol-soluble non-cell-wall material in the work by Hilz et al. (26).

Bilberry DF carbohydrate is composed of pectin, hemicellulose, and cellulose, from which the hemicellulose is mostly xyloglucan. The xylose-to-glucose ratios were 0.14 and 0.41 in whole bilberry and in the press cake, respectively, suggesting removal of non-cell-wall glucose with the juice process and increase in cellulosic and hemicellulosic cell-wall material, which is in line with Hilz et al. (26). The xylose-to-glucose ratio in our study in coarse skin, fine skin, seed, and fine fractions were 0.35, 0.64, 0.57, and 0.35, respectively. The higher xylose-to-glucose ratios in the seed and fine skin fraction in our study indicate the lower proportion of pulp and glucose in comparison with the coarse skin and fine fractions. Higher proportions of glucose in all the fractions indicate that some glucose also originates from the remnant juice, which explains the low xylose-to-glucose ratios in all the fractions of the present study compared with the work of Hilz et al. (26). The xylose-to-glucose ratio in seeds was 1.19 in the work of Hilz et al. (26), and the seeds in their study were separated after alcohol extraction, resulting in better separation from pulp and juice than in the present study. However, higher xylose-to-glucose ratio in bilberry (0.83) (26) can also indicate the presence of cell-wall xyloglucans, as suggested by Vicente et al. (54).

Interactions of the very insoluble DF analysed here as a sum of SSM and SIM with colonic bacteria remain to be studied. We have previously shown that recalcitrant lignin-rich fractions of brewer's spent grain are to a small extent metabolised in an *in vitro* colon model to, for example, 4-methyl catechol (55, 56), and ferulic acid together with its metabolites such as hydroxylated phenylpropionic and acetic acids (57) has been suggested to be one mediator of health effects of cereal DF (58). Bilberry phenolics are mainly anthocyanins, which can be converted to their specific benzoic acid derivatives (59, 60). Therefore, it can be anticipated that bilberry would give distinctly different outcome after colonic conversions than other DF sources, such as cereal grains. There are very few studies about the physiological effects after using bilberry press cake as a source of DF. However, Håkansson et al. (61) could reduce inflammation in chemically induced colitis in rats by combination of probiotics and bilberry skins or rye bran, which could be used for amelioration of intestinal inflammation. Furthermore, Bränning et al. (62) used also bilberry skins in a rat study. The bilberry-skin-diet increased the faecal bulking and transferred colonic fermentation of DF towards the distal colon, in which the proportion of propionic acid was higher than in the control group. Bränning et al. (62) described also a disease activity index, which correlated negatively with the formation of several carboxylic acids, presumably reflecting conversion of polymeric and monomeric phenolic compounds by the gut microbiota (63). The press cake in the present study contained more non-carbohydrate fibres and cell-wall polysaccharides than has been reported for skins in the studies by Bränning et al. (62), reflecting difference in the efficacy of modern juice pressing processing.

In conclusion, the insoluble and non-carbohydrate nature of DF of bilberries is quite different from that in, for example, grains and vegetables, which are the major sources of DF in the Finnish diet (8). The current average DF intake in Finland is 22 g (women) or 23 g (men). To increase the daily intake of DF to the recommended minimum level of 25 g/day, consumption of 100–133 g bilberries or 5.4–7.2 g dry bilberry press cake is recommended. This would increase the amount of very recalcitrant insoluble DF and bring anthocyanins as co-passengers, thus diversifying DF intake. Non-carbohydrate DF may have distinct physiological effects, the health relevance of which calls for further studies.
